# Disassembly Properties of Cementitious Finish Joints Using an Induction Heating Method

**DOI:** 10.3390/ma8052433

**Published:** 2015-05-08

**Authors:** Jaecheol Ahn, Takafumi Noguchi, Ryoma Kitagaki

**Affiliations:** 1 Department of Architecture, Dong-A University, 550 Beon-gil Saha-gu, Busan 604-714, Korea; 2 Department of Architecture, the University of Tokyo, Hongo 7-3-1, Tokyo 113-8654, Japan; E-Mails: noguchi@bme.arch.t.u-tokyo.ac.jp (T.N.); ryoma@bme.arch.t.u-tokyo.ac.jp (R.K.)

**Keywords:** induction heating, cementitious joint, mortar, steel fiber, conductive resistor, disassembly property

## Abstract

Efficient maintenance and upgrading of a building during its lifecycle are difficult because a cementitious finish uses materials and parts with low disassembly properties. Additionally, the reuse and recycling processes during building demolition also present numerous problems from the perspective of environmental technology. In this study, an induction heating (IH) method was used to disassemble cementitious finish joints, which are widely used to join building members and materials. The IH rapidly and selectively heated and weakened these joints. The temperature elevation characteristics of the cementitious joint materials were measured as a function of several resistor types, including wire meshes and punching metals, which are usually used for cementitious finishing. The disassembly properties were evaluated through various tests using conductive resistors in cementitious joints such as mortar. When steel fiber, punching metal, and wire mesh were used as conductive resistors, the cementitious modifiers could be weakened within 30 s. Cementitious joints with conductive resistors also showed complete disassembly with little residual bond strength.

## 1. Introduction

The building production technologies that have been realized to date include those for the composition of building materials and structural members, as well as the simplification of assembly processes. However, because of the low disassembly properties of individual materials and parts, efficient maintenance and upgrading of buildings during the course of their lifecycles have presented challenges. Furthermore, the reuse and recycling processes during the demolition of buildings also involve numerous problems from the perspective of environmental technology [1,2]. In Japan, the impurity contents of finishing materials have been greater than 10% of total concrete waste, even if the preceding collection of demolished finishing materials was conducted at demolition site of reinforced concrete structure buildings. This affects the quality of building material recycling as a result [3].

In this study, induction heating was used to determine the disassembly properties of a cementitious joint widely used to join building members and materials. The induction heating technique has high energy efficiency [4,5,6,7,8]. Hence, it is expected to provide local heating only for building members and materials that are in contact. Therefore, this technique is highly regarded as an environment-friendly technique for a forward-backward process. In particular, because wire mesh and rebar are widely used as the conductive materials of the heating source for induction heating in existing buildings, they are judged to be capable of facilitating the easy specification of disassembly properties without the deterioration of the assembly properties.

Therefore, the purpose of this study was to specify disassembly properties that enable field disassembly while maintaining assembly properties such as workability and bond strength, using a conductive resistor at a cementitious joint. In addition, basic data for the development of an optimal forward-backward process production system were proposed by selecting a conductive resistor with outstanding temperature elevation characteristics in response to induction heating.

## 2. Separation Model for Cementitious Joints during Induction Heating

Cementitious materials are likely to decrease in strength because of the dehydration and metamorphosis of the inner hydrate and the accompanying increase in the pore volume when subjected to heating at 300 °C [9,10]. If the cement paste, the main component of mortar, is dehydrated and weakened by heating the adhered mortar, the attached material and adhesive mortar can be efficiently separated. In particular, the induction heating method [11] can be used to selectively heat only the desired part with outstanding heat efficiency. Hence, it is deemed capable of improving the cementitious material’s temperature elevation characteristics in the most economical way.

**Figure 1:**
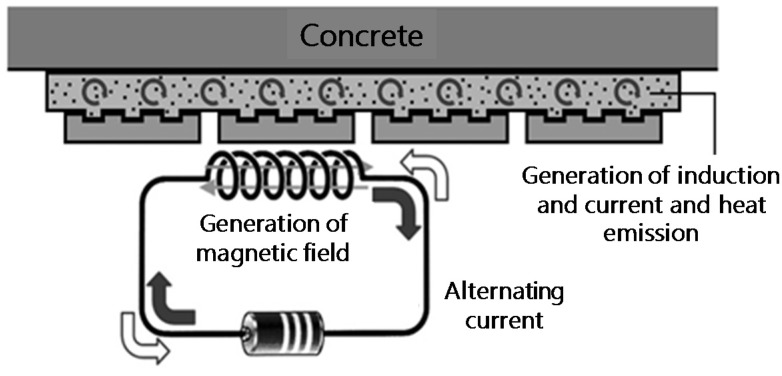
Separation mechanism of a cementitious joint using induction heating method.

**Figure 2:**
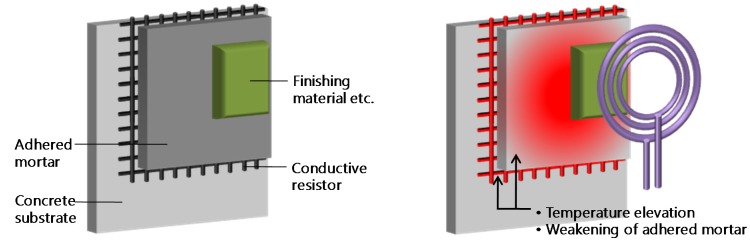
Separation model of finishing items.

Therefore, if a conductive resistor such as steel fiber or a wire fabric is mixed into the adhered mortar, the disassembly property can be specified, while maintaining the assembly properties of the mortar-like bond strength and workability. Figure 1 shows the disassembly mechanism of the induction heating method, and Figure 2 shows a separation model of the finishing items attached as cementitious material.

## 3. Temperature Elevation Characteristics of Conductive Resistors under Induction Heating

### 3.1. Overview of Experiment

In this study, a conductive resistor with high energy efficiency was selected by analyzing the temperature elevation characteristics of induction heating according to the type of conductive resistor. The efficiency of the induction heating was anticipated to be significantly affected by the cross-sectional area of the conductive resistor, and the wire fabric was expected to vary according to the adhered shape of the contact points of the horizontal and vertical wires [12]. The temperature elevation characteristics as a function of the radio frequency and heating distance of the conductive resistor were also analyzed.

### 3.2. Experiment Factors

Wire, stainless metal mesh, and punching metal were used as conductive resistors. After adjusting the radio frequency power to 1, 2.25, and 4 kW, variations in the temperature elevation characteristics according to the type of conductive resistor were analyzed, and the results are listed in Table 1.

**Table 1:** Experimental factors for temperature elevation.

Specimen Symbol	Conductive Resistor	
	Metal Mesh and Punching Metal	Size
F4.0	Wire fabric (zinc plate)	ø1.60 mm × P4.75 mm (4 Mesh)
F10		ø0.55 mm × P1.99 mm (10 Mesh)
S4.0	Stainless fabric	ø1.60 mm × P4.75 mm (4 Mesh)
S10		ø0.53 mm × P2.01 mm (10 Mesh)
Fp0.5–ø3	Steel punching metal	T0.5 mm–ø3 mm × P5 mm (Opening rate 32.4%)
Fp1.0–ø3		T1.0 mm–ø3 mm × P5 mm (Opening rate 32.4%)
Fp1.0–ø5		T1.0 mm–ø5 mm × P6 mm (Opening rate 62.5%)
Sp0.5–ø3	Stainless punching metal	T0.5 mm–ø3 mm × P5 mm
Sp1.0–ø3		T1.0 mm–ø3 mm × P5 mm

Radio frequency power: 1 kW, 2.25 kW, 4.0 kW.

### 3.3. Production of Materials and Specimen

**Figure 3:**
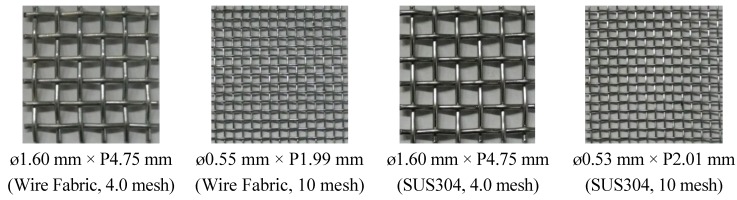
Wire fabric and stainless fabric.

For the metal mesh, a ø1.60 mm × P4.75 mm 4.0 mesh or ø0.55 mm × P1.99 mm 10 mesh of zinc-plated wire fabric was used, where ø and P denote the diameter (mm) and pitch (mm), respectively. For the stainless mesh, a ø1.60 mm × P4.75 mm 4.0 mesh or ø0.53 mm × P2.01 mm 10 mesh of SUS304 (18Cr-8Ni) was used. Figure 3 shows the wire fabric and stainless fabric metal meshes.

**Figure 4:**
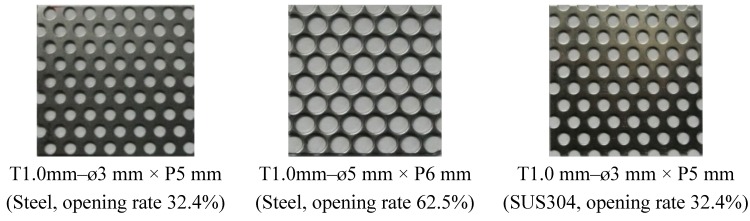
Punching metal.

The punching metal material was the same as that of the metal mesh. For the punching metal, either a steel with a 32.4% (ø3 mm × P5 mm) opening rate and a thickness (T) of T0.5 or T1.0 mm or one with a 62.5% (ø5 mm × P6 mm) opening rate and a thickness of T1.0 mm was used. For the stainless punching metal, a plate with a 32.4% (ø3 mm × P5 mm) opening rate and a thickness of T1.0 mm was used. The hole was punched at the top of a regular triangle. Figure 4 shows the punching metals used in this study.

### 3.4. Induction Heating

**Figure 5:**
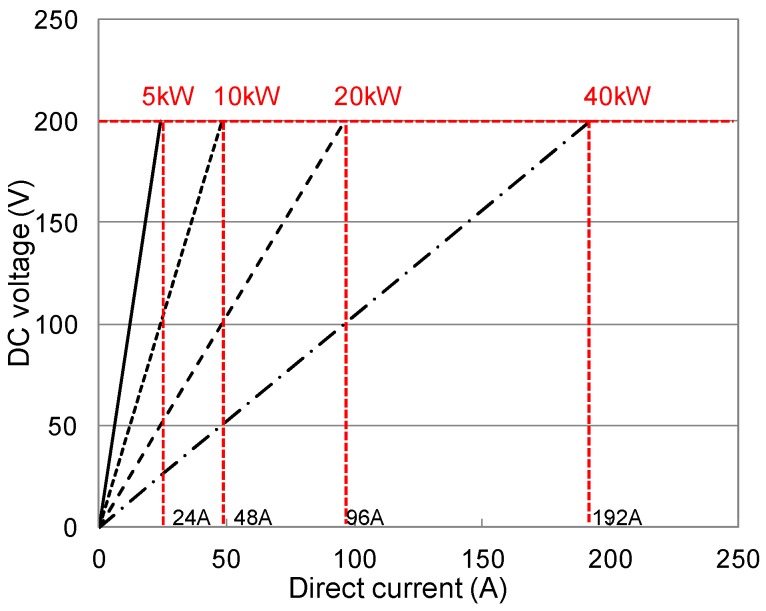
Induction heating power.

An experimental device with a 100 kHz (60 to 120 kHz operating frequency) and 5 kW maximum radio frequency was used for the induction heating. The power was stable within a range of ±2%, as shown in Figure 5. The power was adjusted using the direct current (DC) to DC voltage relationship. For the radio frequency power, an auto-track method was adopted, with a resonance frequency determined by the resonance condenser inside the adapter, as well as the inductances of the power lead and heating induction coil. The heating induction coil had a ø10 mm with an external diameter of ø120 mm. Each temperature measurement was conducted using an infrared thermometer (thermographic camera) in the temperature range of 0 °C–800 °C.

### 3.5. Temperature Elevation Characteristics of Conductive Resistor by Induction

**Figure 6:**
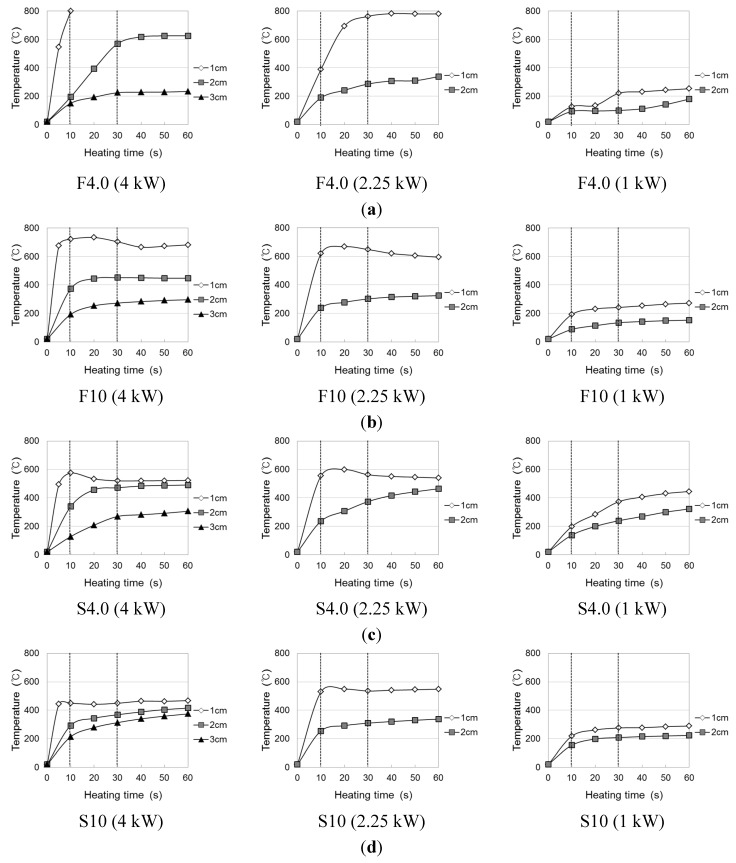
Temperature elevation of induction heating of conductive resistor (Metal mesh). (**a**) Wire fabric of 4 mesh; (**b**) wire fabric of 10 mesh; (**c**) stainless fabric of 4 mesh; (**d**) stainless fabric of 10 mesh.

**Figure 7:**
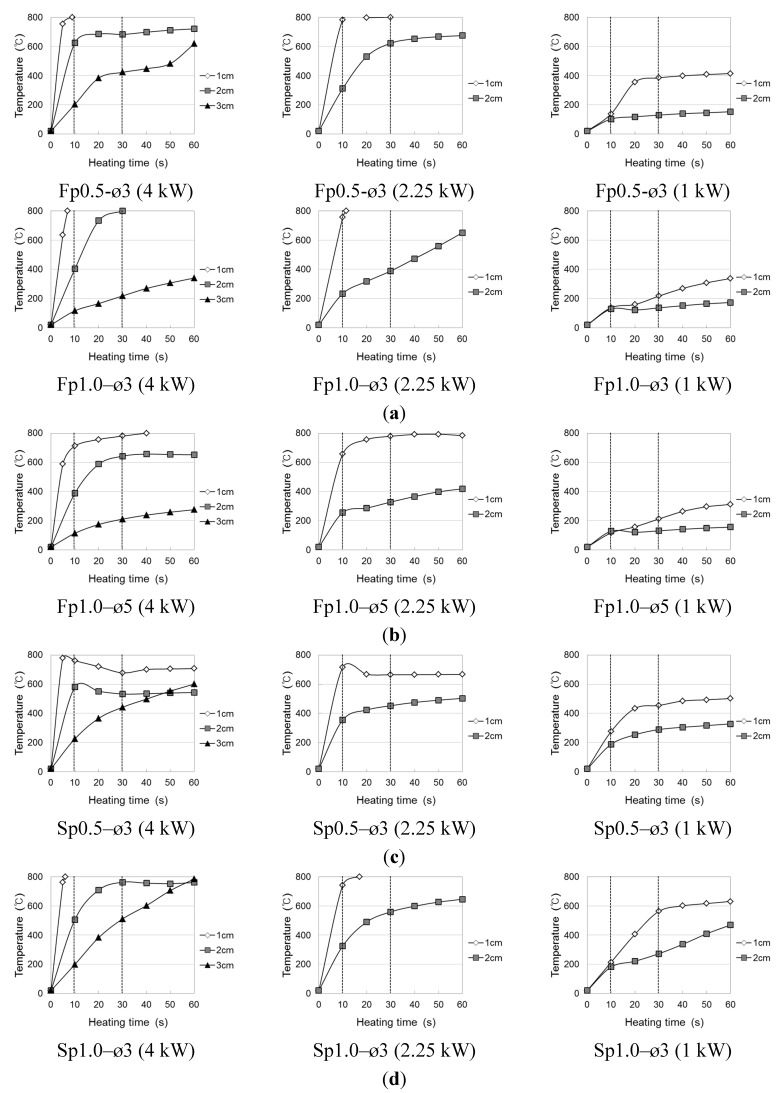
Temperature elevation of conductive resistor (Punching metal). (**a**) Wire punching metal of diameter 3 mm; (**b**) wire punching metal of diameter 5 mm; (**c**) stainless punching metal of thickness 0.5 mm; (**d**) stainless punching metal of thickness 1.0 mm.

The temperature elevation characteristics of the conductive resistors during induction heating are shown in Figure 6 and Figure 7 as a function of the type of resistor (metal mesh and punching metal), power consumption, and heating distance. The experiments were performed with a basic frequency of 100 kHz, and three different power levels were used: 1 kW (DC voltage 100 V × DC current 10 A), 2.25 kW (DC voltage 150 V × DC current 15 A), 4 kW (DC voltage 200 V × DC current 20 A). For temperatures above 800 °C, which could not be measured, the value was set to 800 °C. To measure the overall temperature elevation characteristics, heating gradually progressed toward the inside as a function of time to make heating the outside easier than heating the center. In addition, the temperature elevation characteristics varied depending on the type and cross-sectional area of the conductive resistor.

**Figure 8:**
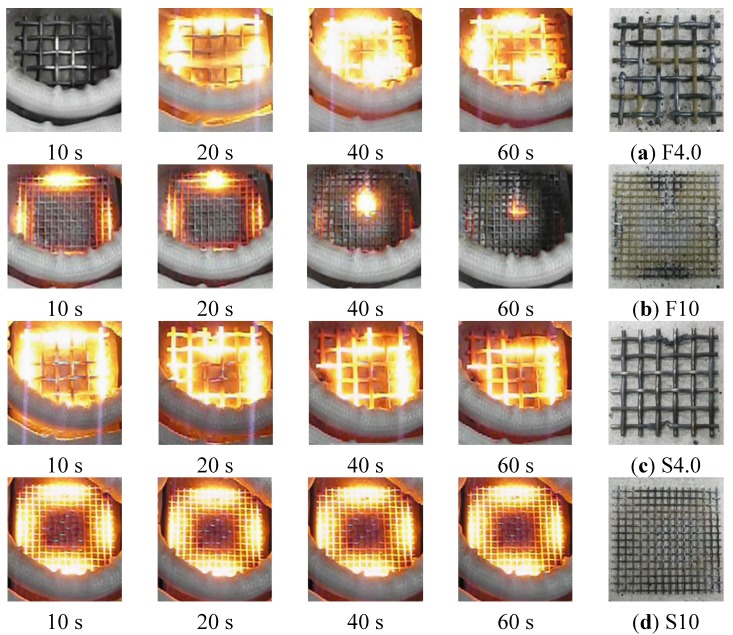

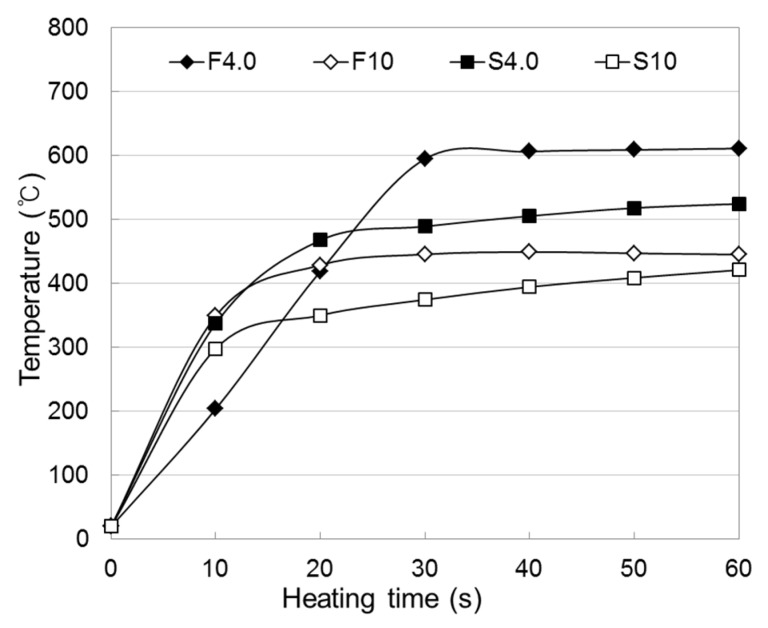
Appearance of heating conductive resistor by induction (metal mesh, 4 kW–2 cm).

At a radio frequency power of 4 kW with a metal mesh as the conductive resistor and an induction heating distance of 1 or 2 cm from the coil to the conductive resistor, the temperature sharply increased with the heating time from 10 to 30 s and thereafter tended to remain constant or slightly decrease. This indicated that the induction heating energy was concentrated in both the wire fabric and stainless fabric (Figure 8; heating distance of 2 cm). Therefore, the function of the circuit that facilitated the induction heating was terminated because the outside, which was heated the fastest, was fractured as it reached the melting point. For this reason, the temperature decreased, while a new circuit was created at the center, where the heat efficiency was relatively low. In particular, in the case of the 10-mesh wire fabric (specimen F10), the highest temperature was the same as that in the case of the stainless fabric, despite the significant decrease in heating area. This was attributed to the low melting point of the zinc plating applied to prevent the wire fabric from corroding, which caused an electrical resistance across the interface of the melted and non-melted parts. Another reason is that the wire fabric may not readily serve as a second circuit for adhesion among the wires because of the melting of the zinc plating. However, the temperature increased gradually when the circuit was not fractured at a distance of 3 cm. By the same principle, when the radio frequency power was 2.25 kW, with a heating distance of 2 or 3 cm, the temperature increased gradually over time. However, when the heating distance was 1 cm, the rates of temperature elevation hardly differed between 4 and 2.25 kW; additionally, as the heating distance gradually increased, the efficiency of the heating by the radio frequency power increased.

The thickness of the conductive resistor affected the temperature elevation characteristics. Temperature increased gradually in the 4-mesh stainless fabric and wire fabric, in which a second circuit was readily created within the cross section by the induction heating as a result of the large cross section of each wire of the metal mesh. Regarding the effect of the material type on the temperature elevation characteristics, the highest temperature was shown by the wire, which, as a magnetic material, exhibited a heat emission reaction by a hysteresis loss through heating by an eddy current. In contrast, the stainless steel showed a more uniform heating tendency at the cementitious joints because of higher thermal conductivity.

**Figure 9:**
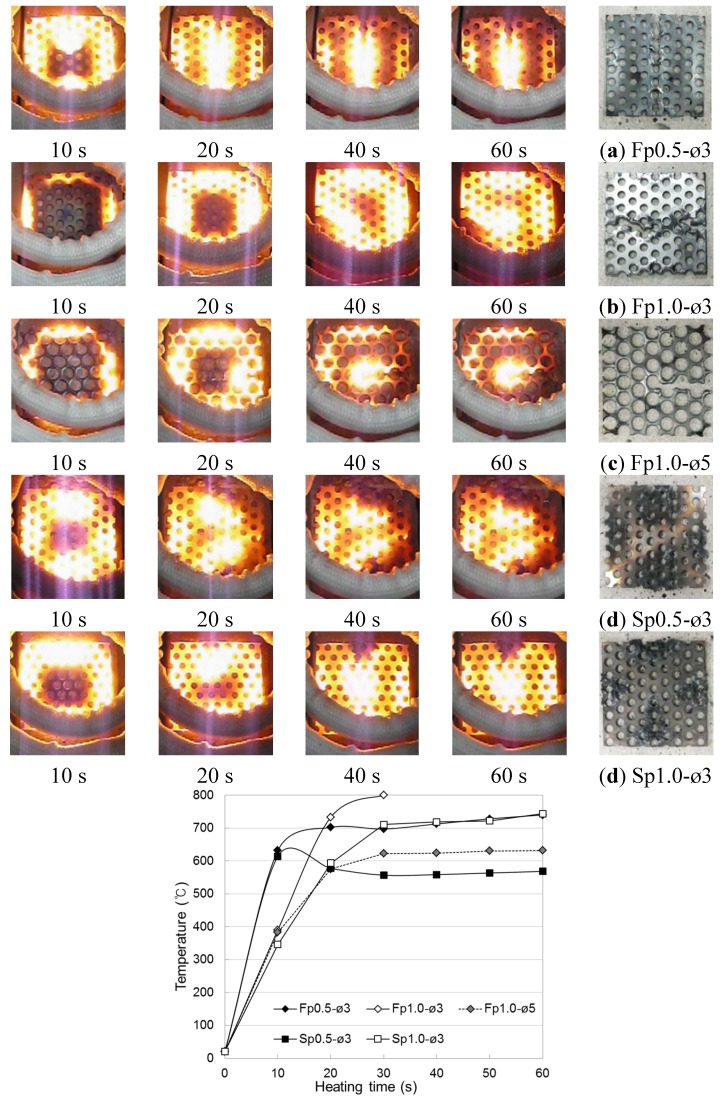
Appearance of heating conductive resistor by induction (punching metal, 4 kW–2 cm).

In the case of the punching metal (Figure 9), the rate of temperature elevation was exceedingly high compared with that in the case of the metal mesh. Hence, the rate could not be measured because the temperature reached 800 °C within 10 s at the 4-kW heating condition, with the difference increasing with a decrease in the heating distance. This was because the contact point of the punching metal in a wire fabric is large, whereas the contact area of a horizontal line and vertical line is small. Hence, a second eddy current easily occurs as a result of induction heating. Although the resistor thickness had hardly any effect, in case of the 0.5-mm stainless punching metal, the temperature decreased because of a fracture after reaching the highest temperature. As for the difference caused by the punching metal opening rate, the rate of temperature elevation was slightly lower at ø5 mm (opening rate 62.5%) than at ø3 mm (opening rate 32.4%). However, in the case of the 1-kW radio frequency power, the temperature could not exceed 400 °C, where the weakening of the cementitious material was anticipated, even after 60 s of heating.

Therefore, in the cases where the metal mesh and punching metal were used as conductive resistors, 2 cm was judged to be the appropriate distance for the induction coil to allow the conductive resistor to weaken by heating the cementitious joint. If the energy efficiency and temperature elevation characteristics are considered, the appropriate power would be 2.25 kW in the case of a heating distance of 1 cm and 4 kW in the case of a heating distance of 1 to 2 cm.

## 4. Evaluation of Disassembly Properties of a Cementitious Joint during Induction Heating

### 4.1. Overview of Experiment

Experiments were performed to analyze the temperature elevation characteristics of the induction heating and the disassembly performance as a function of the type of conductive resistor. Specifically, experiments were carried out under a condition in which a wire mesh, stainless metal mesh, or punching metal was inserted into adhered mortar as a conductive resistor and one in which steel fiber was mixed into the adhered mortar directly. The disassembly performance of the cementitious joint was determined by the effect of selectively heating (by induction heating) the conductive resistor analyzed in the previous section, and the consequent weakening of the cementitious joint. In addition, the following issues were analyzed in the case of the steel fiber: whether a closed circuit formed depending on the degree of fiber distribution inside, and the effect on the weakening of the adhered mortar of varying the composition using fibers with different thicknesses and lengths. Through the analysis of the adhered mortar using a conductive resistor, integrative evaluations of the assembly and disassembly properties were also conducted. Table 2 lists the experimental factors used to evaluate the assembly properties.

**Table 2:** Experimental factors for evaluation of assembly property.

Specimen Symbol	Conductive Resistor	
	Conductive Resistor Shape	Specification
N	-	-
F4.0	Wire Fabric (zinc plated)	Diameter 1.60 mm × P4.75 mm (4 Mesh)
F10		Diameter 0.55 mm × P1.99 mm (10 Mesh)
S4.0	Stainless fabric (SUS304)	ø1.60 mm × P4.75 mm (4 Mesh)
S10		ø0.53 mm × P2.01 mm (10 Mesh)
Fp0.5–ø3	Steel punching metal	T0.5 mm–ø3 mm × P5 mm
Fp1.0–ø3		T1.0 mm–ø3 mm × P5 mm
Fp1.0–ø5		T1.0 mm–ø5 mm × P6 mm
Sp0.5–ø3	Stainless punching metal	T0.5 mm–ø3 mm × P5 mm
Sp1.0–ø3		T1.0 mm–ø3 mm × P5 mm
SF06–*	Steel fiber 6 mm	*: contents rate (1%, 2%, 3%)
SF13–*	Steel fiber 13 mm	

### 4.2. Production of Materials and Specimens

**Figure 10:**
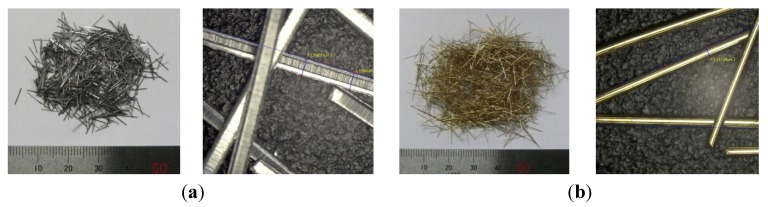
Steel fiber. (**a**) Steel fiber SF06 (= 6 mm); (**b**) steel fiber SF13 (= 13 mm).

The metal mesh and punching metal used as the conductive resistors were the same as those discussed in the previous section. Steel fiber 1 (SF06), as shown in Figure 10, was manufactured by shear-machining a thin steel plate produced by cold rolling into the shape of a fiber with the length of 6 mm and cross section of 0.25 mm per side for the aspect ratio of 24. The dimensions of steel fiber 2 (SF13) were a length of 13 mm and diameter of 0.16 mm for an aspect ratio of 81. The specific gravity (7.85 t/m^3^) was the same. Steel fiber 2 was brass-plated to prevent corrosion.

The adhered mortar into which the wire fabric and punching metal were inserted was produced by mixing water (W) and cement (C) at a W/C ratio of 35%, with 10% polymer to prevent the hardware from sinking (Table 3).

**Table 3:** Combined ratio of adhered mortar mixed with steel fiber.

Steel Fiber Volume Mixed Rate (*V_f_*)	Cementitious Binder (kg)					
	Water	Cement	Polymer	Antifoaming Agent	Thickener	Chemical Agent
0.0	0.35	1	0.1	0.001	-	-
1.0, 2.0	0.40	1	0.1	0.001	-	-
3.0	0.40	1	0.1	0.001	0.002 *	0.015 **

* Thickener: 0.5 wt·% of volume; ** Chemical agent: high performance air entrain water-reducing agent 1.5% of cement weight.

Steel fiber-mixed mortar has the potential to separate if the mortar does not have the proper fluidity and viscosity as a binder, because it contains a large proportion of steel fiber. Therefore, through preliminary experimentation, the mortar combination that achieved the best fiber distribution property was determined based on the proper viscosity and fluidity. An ordinary mortar with a W/C ratio of 40% was composed at a 1% to 2% mixture rate of steel fiber, whereas a ball of fiber was created by the sudden deterioration and separation of the material when steel fiber was mixed in at a rate of 3%. The fluidity and plastic viscosity were adjusted using a thickener and a high-performance, air-entrained, and water-reducing agent, and excluding the use of a mineral admixture to minimize the effect on strength. The particle size of the fine aggregates was adjusted to 2.5 mm or less.

### 4.3. Experimental Method

The induction heating device and methods were the same as those described in the previous section. To analyze the assembly and disassembly properties of a cementitious joint with a conductive resistor, a tensile bond strength test was performed on a specimen at 28-day age before and after induction heating. For each specimen, 40 mm × 40 mm × 10 mm of adhered mortar was piled at the center of a mortar substrate of 100 mm × 100 mm × 20 mm.

**Figure 11:**
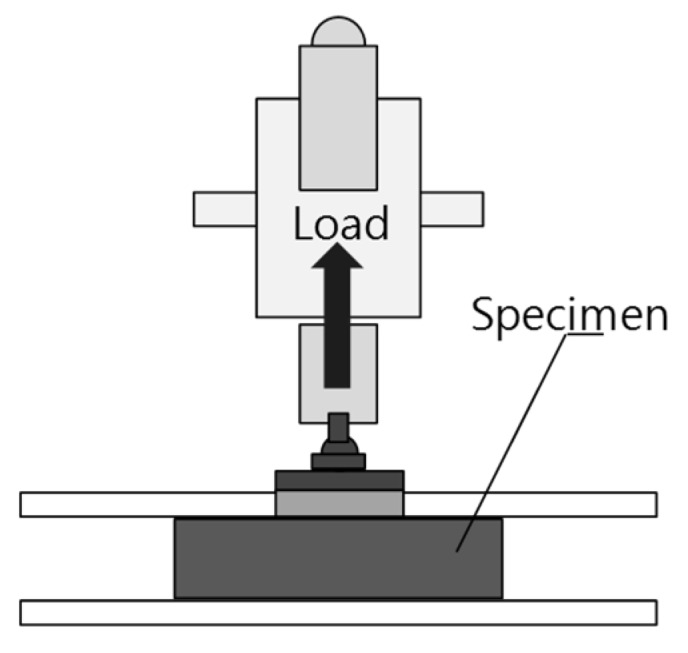
Tensile bond strength test.

Under each condition, conductive resistors were installed in the middle of the adhered mortar. For the test, the adhered mortar surface was made even with the use of sandpaper to ensure that the tensile load would be applied vertically. Subsequently, the test was carried out after the resistor was attached to the device with the use of epoxy resin and left undisturbed for 24 h. The specimen shape and test device are shown in Figure 11.

**Figure 12:**
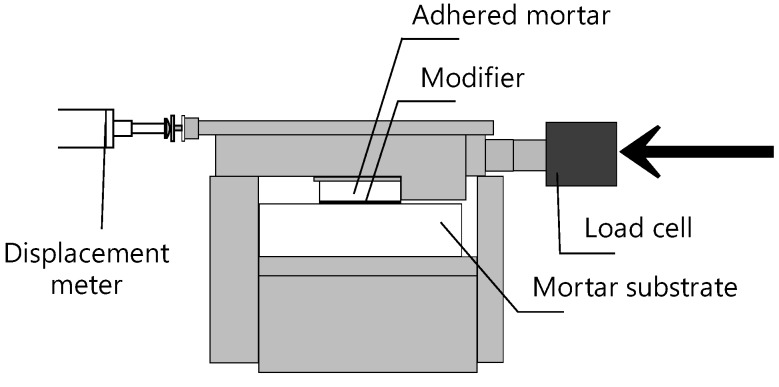
Shear bond strength test.

Shear bond strength test was performed on a specimen at 28-day age before and after induction heating. The specimen shape was the same as that in the tensile bond strength test; the test device is shown in Figure 12. The shear loading and adhered mortar displacement were measured using a load cell and displacement meter, respectively.

The pore-size distribution was measured with the use of a mercury intrusion porosimeter (MIP) to assess the weakening of the cementitious joint by induction heating. Vacuum deaeration was carried out for 2 weeks on each specimen in contact with a conductive resistor after it was taken from the specimen and cooled to room temperature after heating. Additionally, the pore-size distributions were obtained using the MIP at low and high pressures.

### 4.4. Analysis of Assembly Property of Cementitious Joint with Conductive Resistor

**Figure 13:**
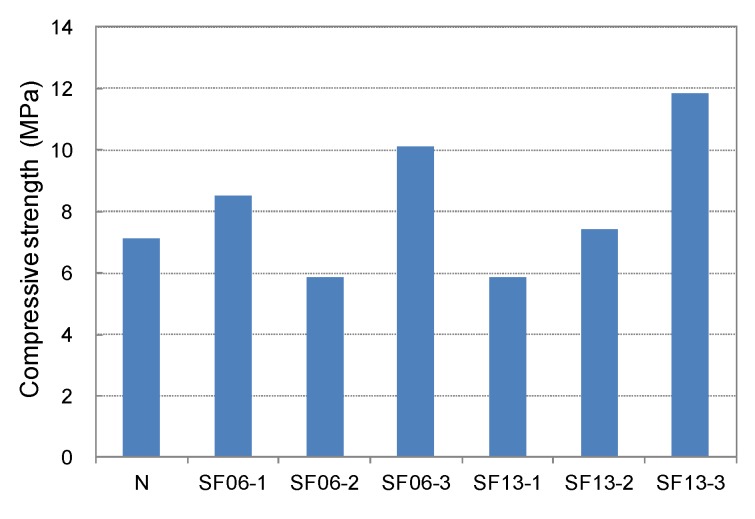
Compressive strength of steel fiber mortar.

**Figure 14:**
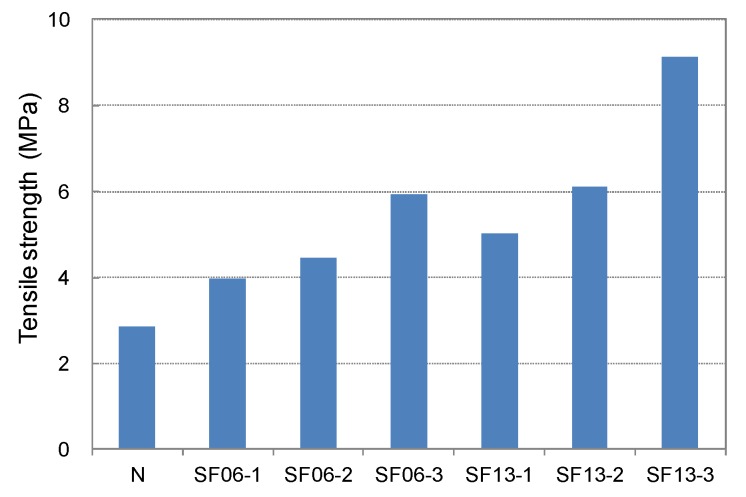
Tensile strength of steel fiber mortar.

**Figure 15:**
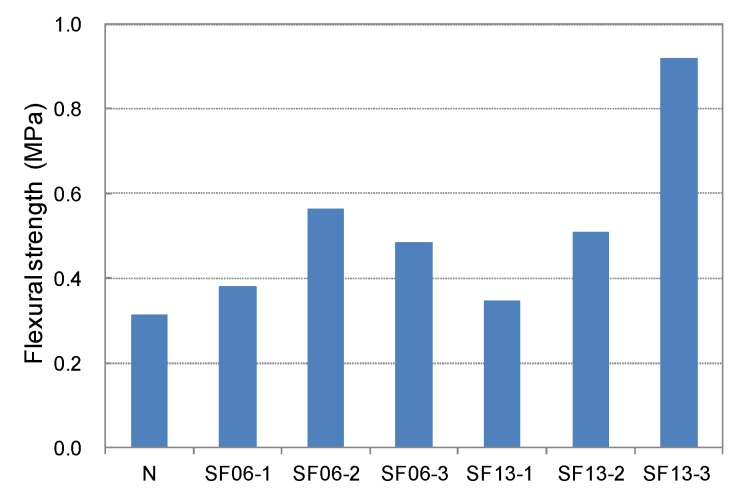
Flexural strength of steel fiber mortar.

**Figure 16:**
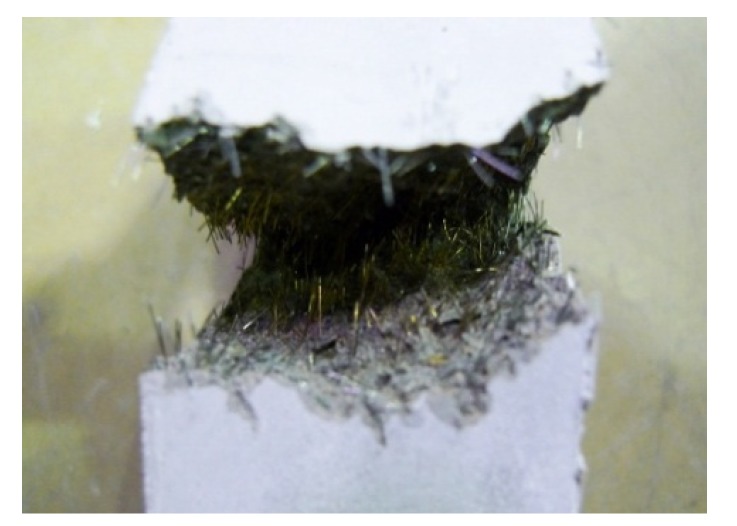
Fractual surface of fiber reinforced mortar.

The compressive, tensile, and flexural strengths of the conductive mortar with steel fibers at 28-day age are shown in Figure 13, Figure 14, Figure 15 and Figure 16, respectively. Compared with SF06 (6-mm-long steel fiber), all of the strengths of the 13-mm-long SF13 were superior, and the tensile and flexural strengths increased significantly with an increase in the mixed fiber. Judged on the basis of its strength, SF13 showed the best assembly properties. Hence, SF13 could be used to produce conductive mortar with outstanding assembly properties by achieving a fluidity and material separation resistance, while improving the strength based on the use of admixtures to promote adhesion between the fiber and matrix.

**Figure 17:**
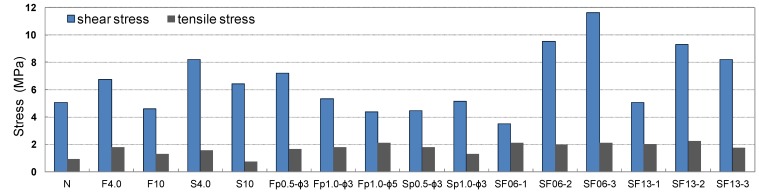
Shear and tensile bond strengths of cementitious joint using a conductive resistor.

**Figure 18:**
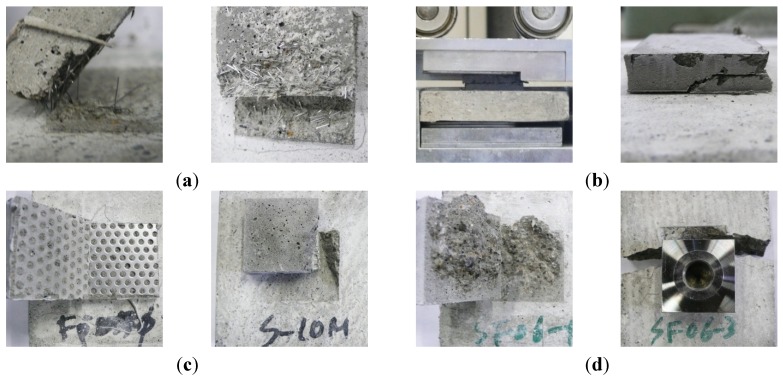
Shapes of bond fracture of cementitious joint using a conductive resistor. (**a**) Improvement of bond strength by mixing of steel fiber (shear); (**b**) shear fracture by metal mesh and punching metal; (**c**) fracture near punching metal (tensile) and metal mesh (shear); (**d**) exfoliation and fracture of mortar substrate (tensile).

The results of the shear and tensile bond strength tests conducted by inserting various types of conductive resistors are shown in Figure 17. In most cases, the strengths were similar to or higher than those obtained without inserting a conductive resistor. The shear bond strength was relatively low in the case of the punching metal, which had the widest separation area on both sides of the adhered mortar. The bond strength also improved when steel fibers were inserted, as shown in Figure 18a,d. Shear and tensile fractures occurred at the interface of the metal resistor, mostly when fabric and punching metal were used, as shown in Figure 18b,c. Specifically, in the case of the wire fabric, separation often occurred at both the adhered mortar-resistor and mortar-mortar substrate interfaces. However, in the case of the tensile bond strength, the adhered mortar-mortar substrate interfaces separated in all the specimens except for the case of Fp1.0-ø3 mm. Therefore, the assembly properties were satisfactory when a conductive resistor was inserted.

### 4.5. Evaluation of Disassembly Properties through Induction Heating of a Cementitious Joint with a Conductive Resistor

#### 4.5.1. Changes in Surface Temperature of Mortar Mixed with Steel Fiber through Induction Heating

**Figure 19:**
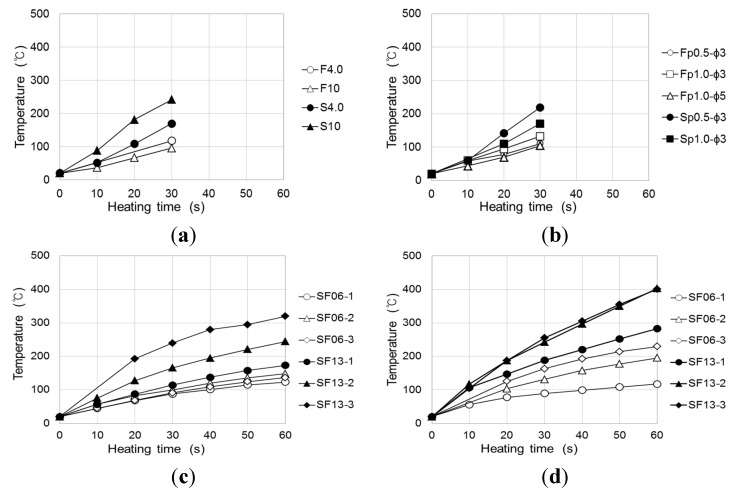
Temperature elevation characteristics of induction heating of adhered mortar to which conductive resistor is mixed. (**a**) Metal mesh (2.25 kW); (**b**) punching metal (2.25 kW); (**c**) steel fiber (2.25 kW); (**d**) steel fiber (4.0 kW).

Figure 19 shows the measurement results for the surface temperature of the adhered mortar during induction heating at a heating distance of 1 cm from the conductive resistor and a frequency power of 2.25 kW. The results show outstanding temperature elevation characteristics in the conductive resistor induction-heating experiment.

Consequently, stainless steel (SUS304), in which uniform cracks may occur, showed slightly higher temperature elevation characteristics for the adhered mortar, depending on the type of resistor material, indicating that this material heats adhered mortar efficiently. The values did not significantly differ irrespective of whether metal mesh or punching metal was used. However, in the former case, the 10 mesh, which was evenly distributed within the mortar, showed an even higher efficiency in the heating mortar despite the thinness of the wire. In the case of the steel fiber, although the temperature increased with the duration of the induction heating, it did not depend on the radio frequency power as it did with the metal mesh or punching metal. The temperature elevation characteristics of SF06 (6-mm-long steel fiber) did not differ significantly with the composition at a power condition of 2.25 kW. However, it was significantly affected by the mixed fiber ratio at 4 kW. In contrast, SF13 showed outstanding overall temperature elevation characteristics compared with SF06. In the case of SF13-3, a mortar temperature elevation of ~250 °C could be obtained through 30 s of heating at 2.25 kW. However, in the case of the steel fiber, the linked condition among the fibers, depending on the internal distribution, significantly affected the efficiency of the induction heating. Therefore, the temperature elevation error was slightly larger compared with those for the wire fabric and punching metal.

#### 4.5.2. Changes in Pore Structure of the Cementitious Joint

**Figure 20:**
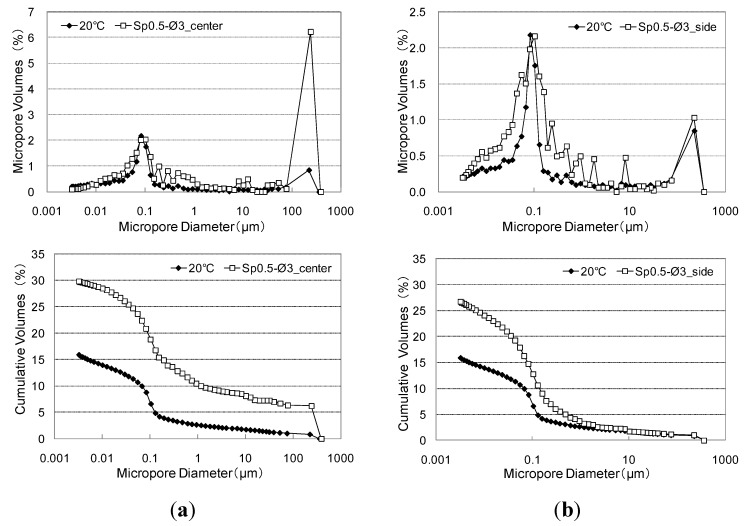
Changes in the pore structure of cementitious joint through induction heating. (**a**) Sp0.5–ø3_2.25 kW (center); (**b**) Sp0.5–ø3_2.25 kW (side).

Figure 20 shows the results of the MIP test for changes in the pore structure due to elevated temperature of a cementitious joint with punching metal. Overall, high peak values appeared near 0.1 μm, and the cumulative amount of pores in the adhered mortar increased the volume by ~10% as a result of induction heating. However, because induction heating only causes intensive heating on the surface of parts in contact with the conductive resistor, this method does not ensure uniform heating throughout the adhered mortar over a short time. Consideration of the size of the porosimeter specimen (a hexahedron of 5 mm) revealed that C–S–H and calcium hydrogen were dehydrated by the high temperature at the surfaces in contact with the conductive resistor. In turn, this also increased the amount of macropores in the 1-μm range to some extent, although the rate of increase in the amount of fine pores in the range of 0.01–0.5 μm by heating below 300 °C was higher. Therefore, the peak value increased, and the number of fine pores decreased when the fine pore diameter increased with increasing dehydration in contrast with totally heated mortar [13].

#### 4.5.3. Evaluation of Disassembly Property through Residual Bond Strength after Induction Heating

**Figure 21:**
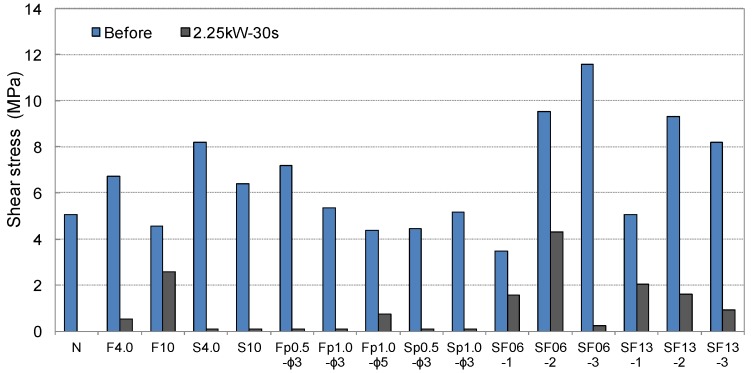
Residual shear bond strength after induction heating of the cementitious joint using a conductive resistor.

**Figure 22:**
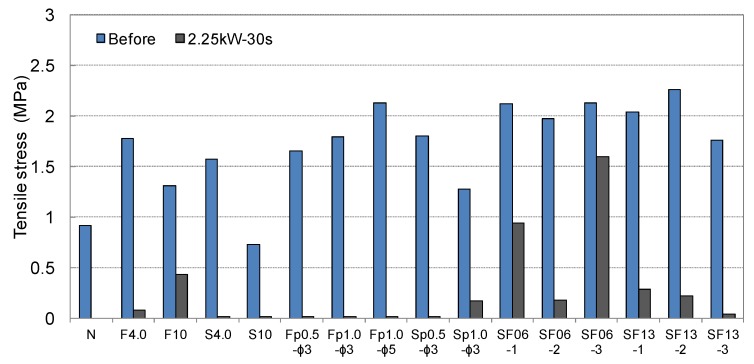
Residual tensile bond strength after induction heating of the cementitous joint using a conductive resistor.

**Figure 23:**
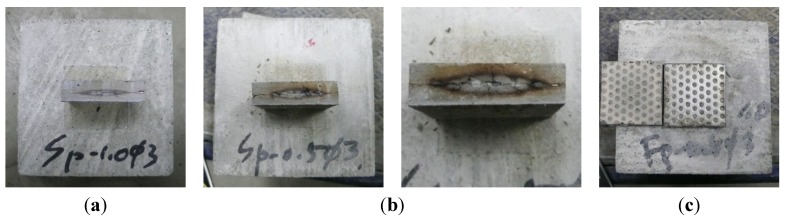
Heating of conductive resistor and weakening of the cementitious joint through induction heating. (**a**) Sp1.0–ø3 (2.25 kW, 30 s); (**b**) Sp0.5–ø3 (2.25 kW, 40 s); (**c**) Fp1.0–ø3 (2.25 kW, 30 s).

Figure 21 and Figure 22 show the residual bond strength after the induction heating of a cementitious joint with a conductive resistor. As a result of induction heating for 30 s with 2.25 kW of power, the bond strength of the joint decreased significantly as a result of the selective heating of the conductive resistor, and the disassembly was the most pronounced when using a punching metal or metal mesh. The surface temperature of the adhered mortar was 100 °C–250 °C, which was slightly low for the cementitious material to be weakened (Figure 19). However, as shown in Figure 23a,b, carbonization and cracks occurred near intensively heated conductive resistors as result of the high-temperature heating. Therefore, in the majority of cases, separation occurred at the interface of the mortar substrate and adhered mortar. Thus, separation often occurred because of local fracturing of the weakened mortar near the punching metal (Figure 23c).

When the metal mesh was used as the conductive resistor, the stainless fabric, which was heated throughout by induction heating, showed an outstanding separation ability compared with the wire fabric. Additionally, separation occurred under no external load immediately after the majority of the specimens were heated, just as when the punching metal was used. In particular, with the punching metal, the temperature elevation characteristics of the metal part itself were similar to those in the metal mesh. However, the punching metal was assumed to undergo thermal expansion. Thus, the adhered part readily weakened because it had a wide area of heat emission. In addition, the steel fibers were assumed to serve as a second circuit when they were distributed and linked to one another inside the matrix, and they demonstrated heat emission characteristics by induction heating. However, in the case of SF06, the bond strength error after heating appeared to be large, and no weakening of the adhesion occurred in some specimens. Hence, the stability of the heating efficiency was assumed to depend on the internal distribution of the fibers. In contrast, SF13 mixed at 2% to 3% showed an outstanding heating efficiency and disassembly. Therefore, the critical mixture rate at which the internal distribution of steel fibers formed a percolation structure was ~2% in the case of SF13. The steel fibers within the joint could be readily separated by the difference in the thermal expansion values of the directly heated steel fiber and mortar and by the weakening of the mortar near the steel fibers.

## 5. Conclusions

By reviewing the temperature elevation characteristics of conductive resistors, the following conclusions were reached about the assembly properties of cementitious joints and their disassembly via induction heating:

(1) Cementitious joints with conductive resistors showed assembly properties equivalent to or more outstanding than those of ordinary finishing items for joint mortar.
(2) Among the conductive resistors, stainless steel showed more uniform temperature elevation characteristics than wire. Furthermore, punching metal readily caused internal eddy currents and showed the most pronounced disassembly properties by the weakening of cementitious joints.
(3) The temperature elevation and weakening of cementitious joints by induction heating were significantly affected by the radio frequency power from the heating induction coil and the distance of the conductive resistor from the heating induction coil.
(4) Conductive resistors could be selected in many ways, depending on the shape and environment of the joint material and the joint shape in each part of a building. The most appropriate conductive resistors for mortar-weakening by thermal conduction were those with at least 10-mesh wire intervals; no deterioration of the adhesion strength was observed when metal mesh was used. In the case of punching metal, a wide interface presumably formed between the adhered mortars, which thus undermined the assembly performance. Therefore, the use of punching metal with diameters of ø5 mm or greater was deemed preferable for the assembly and disassembly properties. Additionally, when SF13 was mixed at a rate of 2% or more, the disassembly performance was outstanding.
(5) Cementitious joints could be disassembled by manual induction heating at 2.25 kW for 30 s. In this case, 1659 W·h disassembly energy was used and 0.921 kg-CO_2_ emission was estimated by using the IH method. Moreover, because such joints have the advantage of being reusable by completely disassembling the materials and parts, induction heating is an appropriate method for disassembling parts that require recycling and the reuse of high-priced materials.
